# Hemangiopericytoma in the Olfactory Groove: A Rare and Unusual Presentation

**DOI:** 10.7759/cureus.1875

**Published:** 2017-11-25

**Authors:** Raghav Gupta, Justin M Moore, Kai Miller, Griffith R Harsh

**Affiliations:** 1 Surgery (division of Neurosurgery), Beth Isreal Deaconess Medical Center; 2 Neurosurgery, Stanford School of Medicine

**Keywords:** hemangiopericytoma, olfactory groove, radiological assessment, histopathology, differential diagnosis

## Abstract

Intracranial hemangiopericytomas (HPCs) are solitary fibrous tumors of the smooth muscle and the mesenchymal origin. While meningiomas located within the olfactory groove are common, an HPC in this location has never been reported previously. Here we describe the rare presentation of a differentiated HPC masquerading as an olfactory groove meningioma in a 33-year-old female presenting with the progressive headaches, anosmia, and visual field disturbances. Following resection, the histopathological analysis confirmed a grade II HPC. A preoperative understanding of the radiographic differences between the meningiomas and HPCs may confirm the treatment planning. An HPC must be considered in the differential diagnosis of the tumors located within the olfactory groove.

## Introduction

Intracranial hemangiopericytomas (HPCs) are rare and constitute less than one percent of all the intracranial tumors [1]. Derived from the mesenchymal origin, the HPCs represent the aberrant growth of Zimmermann’s pericytes, specialized smooth muscle cells, which encircle the capillaries and are essential for regulating the lumen size. The histological examination is often required to differentiate these lesions from radiographically similarly appearing meningiomas. The World Health Organization’s (WHO) 2016 classification of the tumors of the central nervous system (CNS) has combined HPCs and the solitary fibrous tumors within a single category. In this scheme, grade I HPCs represent highly collagenous spindle cell lesions and grade II and grade III HPCs represent differentiated and anaplastic neoplasms, respectively [2].

Given the propensity of these lesions to bleed profusely during the surgical resection and their potential to metastasize distally, early identification is desirable and an aggressive multimodal treatment regimen is warranted. This can include a combination of one or more of the following: preoperative embolization, surgical resection, and/or radiosurgery. The HPCs are most commonly supratentorial dura-associated tumors; they can develop from the dural sinuses, skull-base, falx cerebri, and cerebellar tentorium [1]. While meningiomas within the olfactory groove are fairly common, an HPC in this region has not previously been reported in the literature. Here, we describe the unusual presentation of a histologically confirmed HPC masquerading as an olfactory groove meningioma. We also provide a brief review of the literature on the management of HPCs.

## Case presentation

**Figure 1:**
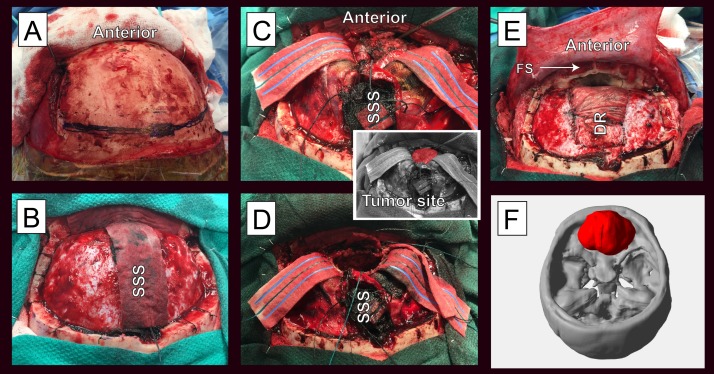
The surgical approach and hemangiopericytoma resection. The surgical approach and resection of the tumor. A: Bicoronal incision has allowed anterior mobilization of a myocutaneous flap, B: The craniotomy has been performed exposing the dura and superior sagittal sinus (SSS), C: The dura has been opened bilaterally and the anterior SSS divided anteriorly to expose the tumor (the insert has the tumor colored in red for clarity), D: The tumor has been completely resected, E: Dural reconstruction utilized a dural synthetic graft (DR) and a pericranial flap was used to seal the frontal sinus (FS) and any anterior cranial fossa (ACF) floor fistulae, F: Three-dimensional (3D) reconstruction of the tumor (red) and skull base.

A 33-year-old Caucasian female presented with progressive headaches, ataxia, anosmia, diminished sense of taste, and visual field disturbances. The ophthalmology examination found papilledema. A comprehensive review of the systems and the physical examination were otherwise negative. The magnetic resonance imaging (MRI) demonstrated an irregularly shaped 6.3 X 4.8 X 4.8 cm intra-dural mass, suggestive of a meningioma arising from the olfactory groove. Intraoperatively, an origin from the olfactory groove was confirmed. Bi-coronal frontal craniotomy accomplished gross total resection of the highly-vascularized tumor (Figure 1). Resection of limited extensions of the tumor through expanded olfactory tract perforations in the olfactory groove necessitated repair of the anterior cranial fossa with a pericranial graft. There were no surgical complications.

Post-operatively, the computed tomography (CT) scan demonstrated removal of the tumor and a small amount of blood in the resection cavity. At discharge, on postoperative day (POD) eight, the patient was neurologically intact to the exam and ambulated independently. She was referred to a radiation oncologist for adjuvant therapy. At her six-week postoperative visit, her previous visual symptoms and headaches had subsided. The analysis of the tumor specimen revealed a spindle cell neoplasm associated with perivascular collagen, positive signal transducer and activator of transcription 6 (stat6) expression, normal mitotic activity (four mitoses per 10 high-powered fields), and no necrosis, all of which were consistent with the World Health Organisation (WHO) grade II lesion. An HPC had not been considered in the differential prior to the surgical resection, given the location of the lesion.

## Discussion

Background

Hemangiopericytomas form a small subset of all intracranial neoplasms and are typically diagnosed in the young males. The surgical management is challenged by the high vasculature nature of these tumors. Their ability to metastasize to other organs, including the lung and liver and their high rates of recurrence following gross total resection (GTR) require multimodal treatment.

Although initially termed “angioblastic meningiomas,” these tumors were reclassified as HPCs by the WHO. Distinguishing an HPCs from a benign meningioma is particularly important as both tumor types are frequently found attached to the dura of the anterior, middle, and posterior cranial fossas. Meningiomas, however, are more common than HPCs, follow a more benign clinical course, and are generally less vascular during the surgery. While a subset of intracranial meningiomas arises from the olfactory groove, an HPC in this location has not been previously reported. Our unique case demonstrates the possibility of occurrence of an HPC in the olfactory groove and warrants effort to distinguish this more aggressive tumor from a benign meningioma, preoperatively.

Hemangiopericytoma vs meningiomas: The radiographic differences

**Figure 2:**
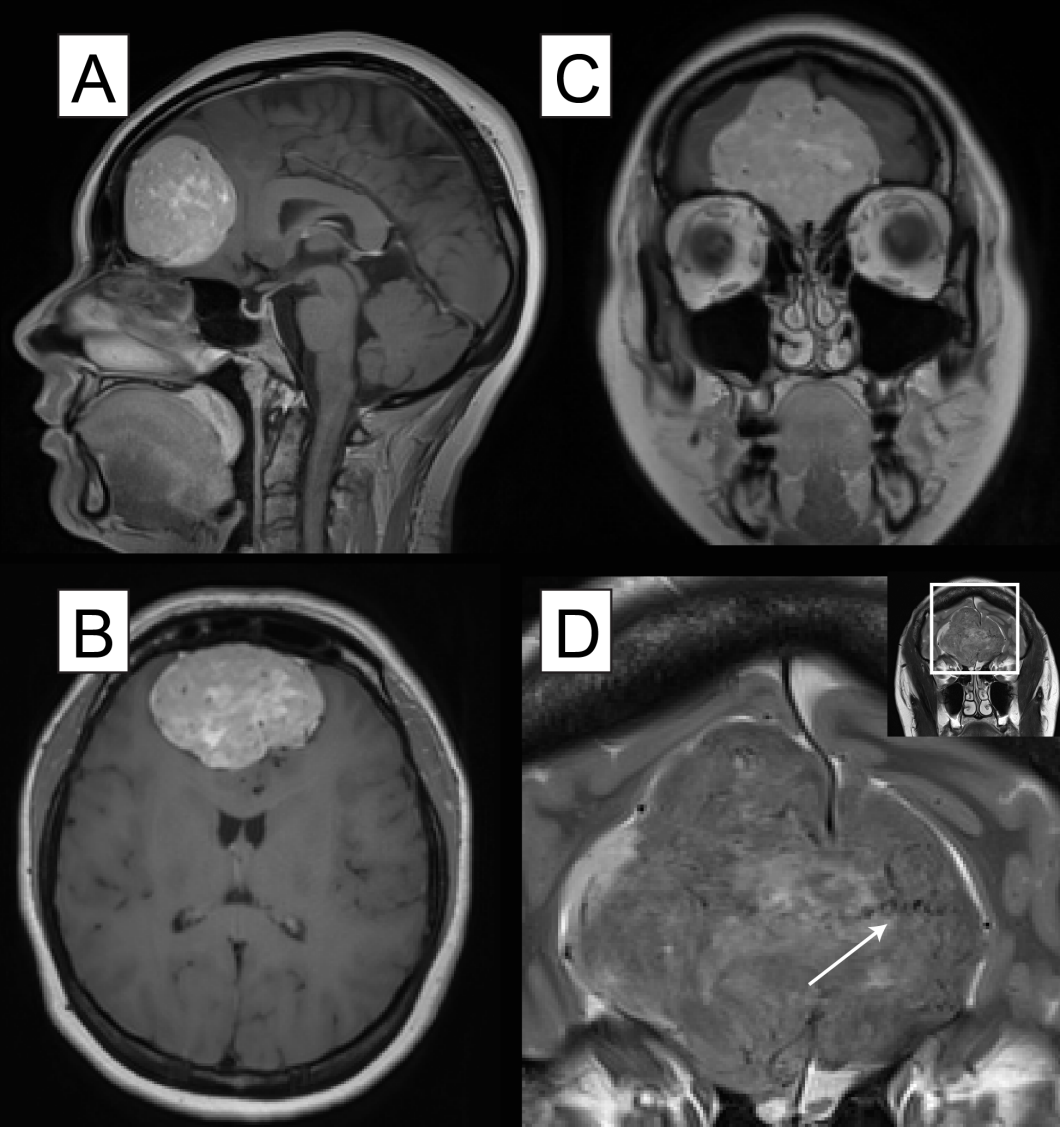
The preoperative magnetic resonance imaging (MRI). The magnetic resonance imaging (MRI) with contrast (A) sagittal (B) axial, and (C) coronal views depict a large contrast-enhancing mass arising from the olfactory groove, D: the MRI T2 weighted image demonstrates corkscrew type flow voids (white arrow), which are consistent with a highly vascular neoplasm.

The histopathological analysis is needed to confirm an HPC as opposed to a meningioma. Nevertheless, the subtle CT and MR imaging features of each entity may help distinguish between them. Chiechi, et al., in their series of 34 HPCs, found that both CT and MR demonstrated a narrow base of dural attachment, in contrast to the broad base of meningiomas. This was observed in our case. The hemangiopericytomas typically have a multi-lobulated, well-marginated contour. Unenhanced CT scans of HPCs may demonstrate adjacent bone erosion; this was found at surgery in our case and necessitated repair of a potential fistula, created by aggressive resection, with a pericranial graft. Unlike meningiomas, the HPCs rarely contain intratumoral calcification or evoke adjacent hyperostosis. The T-1 weighted and T-2 weighted MR images of the HPCs demonstrate heterogeneous, isointense masses with internal vessel flow voids, consistent with the intraoperative finding that these are highly vascular tumors (Figure 2) [3]. The digital subtraction angiography (DSA) can also be used for distinguishing between the meningiomas and the HPCs. A “cork-screw” pattern of intratumoral vessels suggests HPC, while a “spoke-wheel” arterial pattern favors a meningioma. The vascularity of the HPCs and the concomitant increase in the risk of intraoperative hemorrhagic complications may warrant preoperative embolization when safely accessible vessels are seen on preoperative imaging. Additional preoperative measures which provide increased intraoperative hemostatic control in the preparation for the possibility of significant blood loss may also be taken.

Differentiation between the World Health Organisation grade II and grade III intracranial hemangiopericytomas

The recently revised WHO guidelines distinguish grade II and III tumors based on mitotic count and cellular phenotype. The grade II tumors have fewer than five mitoses per 10 high-powered fields. The grade III tumors have five or more mitoses per 10 high-powered microscopic fields, nuclear atypia, high cellularity, or a haphazard, cross-leafed growth pattern. In our case, the pathological analysis revealed a spindle cell neoplasm with low mitotic activity and irregular growth in association with thin vessels and collagen. A WHO grade II differentiated HPC was diagnosed.

Zhou, et al. described the radiological criteria for separating grade II and III lesions, with grade III lesions being irregular in shape and having necrotic, hemorrhagic, and/or cystic components [4]. These features should be kept in mind in cases where an HPC is suspected, as grade III lesions are more aggressive and more likely to metastasize and to recur than are grade II lesions. The gross total resection (GTR) is especially important in these cases. Although adjuvant radiation has been associated with improved outcomes following the surgical treatment in a series containing both grade II and III tumors, its indications for each grade of a tumor independently following either total or incomplete resection is inadequately defined [5].

## Conclusions

Intracranial HPCs are rare, malignant, aggressive neoplasms derived from the smooth muscle cells. A poor clinical prognosis is often associated due to frequent local recurrence of the tumor after gross total resection and distal metastasis. Whereas the olfactory groove is a common site for a meningioma, this is the first reported case of the radiographically similar HPC in this location. As HPCs are highly vascular tumors, the surgical removal can carry significant risk. Appreciation of subtle radiographic differences between HPCs and meningiomas should inform the surgical preparation and performance. The WHO grade II and grade III HPCs should be distinguished from one another as they carry different prognoses and may warrant different treatment regimen.
